# (2*E*)-4-(4-Bromo­phen­yl)-2-{2-[(1*E*)-cyclo­pentyl­idene]hydrazin-1-yl­idene}-3-phenyl-2,3-di­hydro-1,3-thia­zole

**DOI:** 10.1107/S1600536814010897

**Published:** 2014-05-17

**Authors:** Joel T. Mague, Shaaban K. Mohamed, Mehmet Akkurt, Alaa A. Hassan, Mustafa R. Albayati

**Affiliations:** a Department of Chemistry, Tulane University, New Orleans, LA 70118, USA; b Chemistry and Environmental Division, Manchester Metropolitan University, Manchester, M1 5GD, England; c Chemistry Department, Faculty of Science, Minia University, 61519 El-Minia, Egypt; d Department of Physics, Faculty of Sciences, Erciyes University, 38039 Kayseri, Turkey; e Kirkuk University, College of Science, Department of Chemistry, Kirkuk, Iraq

## Abstract

In the title compound, C_20_H_18_BrN_3_S, the cyclo­pentane ring adopts a half-chair conformation. The 4-bromo­phenyl and phenyl rings make dihedral angles of 34.6 (1) and 68.52 (6)°, respectively, with the di­hydro­thia­zole ring. In the crystal, the mol­ecules pack in sheets approximately parallel to (101) which are formed by weak C—H⋯Br inter­actions

## Related literature   

For variuos medicinal applications of thia­zole scaffold compounds, see: Mahajan *et al.* (2008 ▶); Abbs *et al.* (2008 ▶); Chowki *et al.* (2008 ▶); Karabasanagouda *et al.* (2008 ▶); Basavaraja *et al.* (2008 ▶); Bhusari *et al.* (2000 ▶); Basawaraj *et al.* (2005 ▶). For similar structures, see: Akkurt *et al.* (2014 ▶); Mague *et al.* (2014 ▶); Mohamed *et al.* (2013 ▶). For ring conformations, see: Cremer & Pople (1975 ▶).
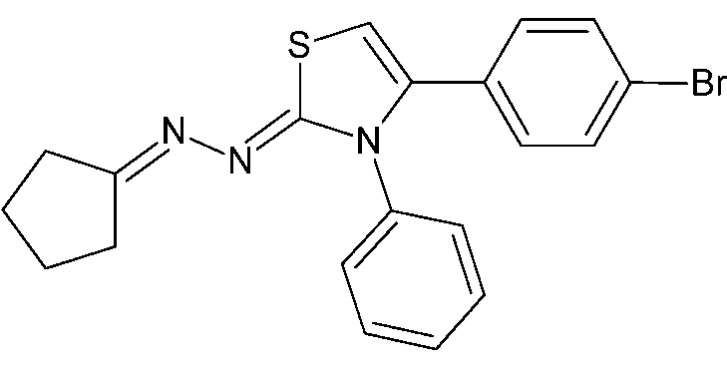


## Experimental   

### 

#### Crystal data  

C_20_H_18_BrN_3_S
*M* *_r_* = 412.34
Monoclinic, 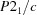

*a* = 12.5079 (3) Å
*b* = 5.5728 (1) Å
*c* = 25.3761 (6) Å
β = 96.8480 (11)°
*V* = 1756.20 (7) Å^3^
*Z* = 4
Cu *K*α radiation
μ = 4.35 mm^−1^
*T* = 100 K
0.10 × 0.08 × 0.02 mm

#### Data collection  

Bruker D8 VENTURE PHOTON 100 CMOS diffractometer
Absorption correction: multi-scan (*SADABS*; Bruker, 2013 ▶) *T*_min_ = 0.80, *T*_max_ = 0.92
12815 measured reflections
3409 independent reflections
2960 reflections with *I* > 2σ(*I*)
*R*_int_ = 0.033

#### Refinement  

*R*[*F* ^2^ > 2σ(*F* ^2^)] = 0.030
*wR*(*F* ^2^) = 0.072
*S* = 1.06
3409 reflections
230 parameters
1 restraint
H-atom parameters constrained
Δρ_max_ = 0.89 e Å^−3^
Δρ_min_ = −0.26 e Å^−3^

### 

Data collection: *APEX2* (Bruker, 2013 ▶); cell refinement: *SAINT* (Bruker, 2013 ▶); data reduction: *SAINT*; program(s) used to solve structure: *SHELXT* (Sheldrick, 2008 ▶); program(s) used to refine structure: *SHELXL2014* (Sheldrick, 2008 ▶); molecular graphics: *DIAMOND* (Brandenburg & Putz, 2012 ▶); software used to prepare material for publication: *SHELXTL* (Sheldrick, 2008 ▶).

## Supplementary Material

Crystal structure: contains datablock(s) global, I. DOI: 10.1107/S1600536814010897/qm2107sup1.cif

Structure factors: contains datablock(s) I. DOI: 10.1107/S1600536814010897/qm2107Isup2.hkl

CCDC reference: 1002511

Additional supporting information:  crystallographic information; 3D view; checkCIF report

## Figures and Tables

**Table 1:** Hydrogen-bond geometry (Å, °)

*D*—H⋯*A*	*D*—H	H⋯*A*	*D*⋯*A*	*D*—H⋯*A*
C17—H17*B*⋯Br1^i^	0.99	3.03	3.828 (2)	138

Symmetry code: (i) 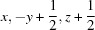.

**Fig. 1.**
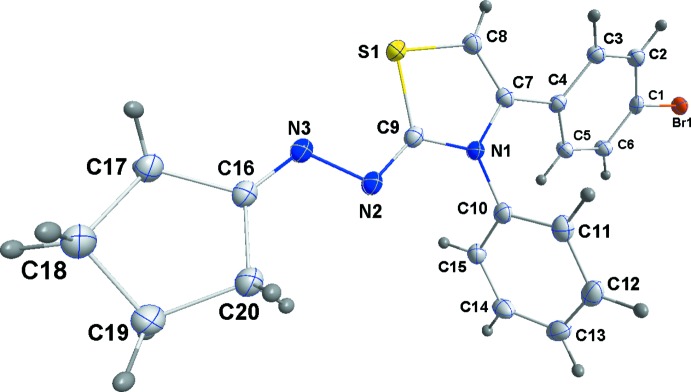
The title compound with 50% probability displacement ellipsoids for non-hydrogen atoms.

**Fig. 2.**
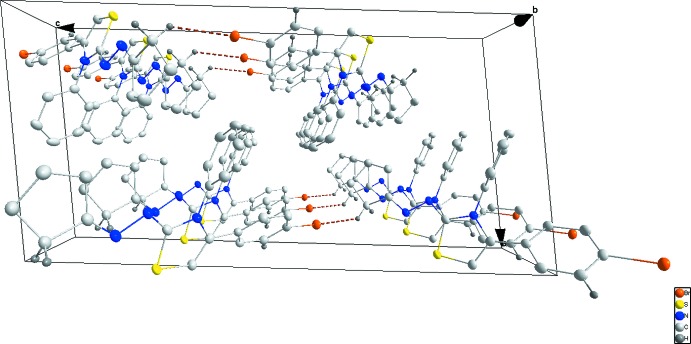
Packing viewed down the b axis with the C—H···Br interactions shown as dotted lines.

## supplementary crystallographic information

### 1. Comment

The anti-microbial activities of substituted thiazoles are well established because they possess the (S—C=N) toxophoric unit (Mahajan *et al.*, 2008). Thiazoles were reported to possess anti-cancer (Abbs *et al.*, 2008), anti-tubercular (Chowki *et al.*, 2008), anti-inflammatory (Karabasanagouda *et al.*, 2008), analgesic (Basavaraja *et al.*, 2008) anthelmintic (Bhusari *et al.*, 2000) and diuretic (Basawaraj *et al*., 2005) activities. Based on these facts and as part of our on-going study we herein report the synthesis and crystal structure of the title compound.

In the title compound (I, Fig. 1), the cyclopentane ring (C16–C20) adopts a half-chair conformation with puckering parameters of Q(2) = 0.375 (3) Å, φ (2) = 270.2 (4) ° (Cremer & Pople, 1975). The dihydrothiazole ring (S1/N1/C7–C9) is essentially planar [max. deviations = -0.002 (2) Å for C8 and C9] and it makes dihedral angles of 34.6 (1) and 68.52 (6)%, respectively, with the 4-bromophenyl (C1–C6) and (C10–C15) phenyl rings. All bond lengths and angles in (I) are normal and comparable with those reported for similar structures (Mohamed *et al.*, 2013; Mague *et al.*, 2014; Akkurt *et al.*, 2014).

In the crystal, the molecules of (I) pack in sheets approximately parallel to (101) which are formed by weak C—H···Br interactions (Table 1, Fig. 2).

### 2. Experimental

A mixture of 1 mmol (233 mg) of cyclopentan-1-one *N*-phenylthiosemicarbazone and 1 mmol (278 mg) of 2-bromo-1-(4-bromophenyl)ethanone in 30 ml e thanol was stirred and refluxed at 350 K. The reaction was monitored by TLC until completion. On cooling, a solid yellow product precipitated which was filtered off and recrystallized from ethanol to furnish yellow crystals, suitable for X-ray diffraction.

### 3. Refinement

H-atoms were placed in calculated positions (C—H = 0.95 - 0.99 Å) and included as riding contributions with isotropic displacement parameters 1.2 times those of the attached carbon atoms. At the conclusion of refinement with all atoms at unit occupancy, the largest difference peak appeared in the vicinity of Br1. Refinement of this as a second component of a disorder of Br1 led to improvement in the results and a more realistic value for U(iso) for Br1. The geometry associated with the minor component (Br1A) suggests that there is a small amount of "*whole molecule*" disorder but since the refined occupancy of Br1A is only 0.02, there is not enough information from the final difference map to reliably position the remainder of the minor component.

### Crystal data

**Table:**

C_20_H_18_BrN_3_S	*F*(000) = 840
*M**_r_* = 412.34	*D*_x_ = 1.560 Mg m^−^^3^
Monoclinic, *P*2_1_/*c*	Cu *K*α radiation, λ = 1.54178 Å
*a* = 12.5079 (3) Å	Cell parameters from 8908 reflections
*b* = 5.5728 (1) Å	θ = 3.5–72.3°
*c* = 25.3761 (6) Å	µ = 4.35 mm^−^^1^
β = 96.8480 (11)°	*T* = 100 K
*V* = 1756.20 (7) Å^3^	Plate, yellow
*Z* = 4	0.10 × 0.08 × 0.02 mm

### Data collection

**Table:**

Bruker D8 VENTURE PHOTON 100 CMOS diffractometer	3409 independent reflections
Radiation source: INCOATEC IµS micro–focus source	2960 reflections with *I* > 2σ(*I*)
Mirror monochromator	*R*_int_ = 0.033
Detector resolution: 10.4167 pixels mm^-1^	θ_max_ = 72.3°, θ_min_ = 3.5°
ω scans	*h* = −14→15
Absorption correction: multi-scan (*SADABS*; Bruker, 2013)	*k* = −6→6
*T*_min_ = 0.80, *T*_max_ = 0.92	*l* = −31→29
12815 measured reflections	

### Refinement

**Table:**

Refinement on *F*^2^	Primary atom site location: structure-invariant direct methods
Least-squares matrix: full	Secondary atom site location: difference Fourier map
*R*[*F*^2^ > 2σ(*F*^2^)] = 0.030	Hydrogen site location: inferred from neighbouring sites
*wR*(*F*^2^) = 0.072	H-atom parameters constrained
*S* = 1.06	*w* = 1/[σ^2^(*F*_o_^2^) + (0.0367*P*)^2^ + 0.942*P*] where *P* = (*F*_o_^2^ + 2*F*_c_^2^)/3
3409 reflections	(Δ/σ)_max_ = 0.004
230 parameters	Δρ_max_ = 0.89 e Å^−^^3^
1 restraint	Δρ_min_ = −0.26 e Å^−^^3^

### Special details

**Table:**

Geometry. All e.s.d.'s (except the e.s.d. in the dihedral angle between two l.s. planes)are estimated using the full covariance matrix. The cell e.s.d.'s are takeninto account individually in the estimation of e.s.d.'s in distances, anglesand torsion angles; correlations between e.s.d.'s in cell parameters are onlyused when they are defined by crystal symmetry. An approximate (isotropic)treatment of cell e.s.d.'s is used for estimating e.s.d.'s involving l.s. planes.
Refinement. Refinement of *F*^2^ against ALL reflections. The weighted *R*-factor *wR* andgoodness of fit *S* are based on *F*^2^, conventional *R*-factors *R* are basedon *F*, with *F* set to zero for negative *F*^2^. The threshold expression of*F*^2^ > σ(*F*^2^) is used only for calculating *R*-factors(gt) *etc*. and isnot relevant to the choice of reflections for refinement. *R*-factors basedon *F*^2^ are statistically about twice as large as those based on *F*, and *R*-factors based on ALL data will be even larger. H-atoms were placed incalculated positions (C—H = 0.95 - 0.99 Å) and included as ridingcontributions with isotropic displacement parameters 1.2 times thoseof the attached carbon atoms. At the conclusion of refinement with allatoms at unit occupancy, the largest difference peak appeared in thevicinity of Br1. Refinement of this as a second component of a disorderof Br1 led to improvement in the results and a more realistic value forU(iso) for Br1. The geometry associated with the minor component (Br1A)suggests that there is a small amount of "whole molecule" disorder butsince the refined occupancy of Br1A is only 0.02, there is not enoughinformation from the final difference map to reliably position theremainder of the minor component.

### Fractional atomic coordinates and isotropic or equivalent isotropic displacement parameters (Å^2^)

**Table:**

	*x*	*y*	*z*	*U*_iso_*/*U*_eq_	Occ. (<1)
Br1	0.85490 (2)	−0.31880 (7)	0.43991 (2)	0.02218 (11)	0.982 (1)
Br1A	0.8563 (12)	−0.201 (4)	0.4313 (4)	0.02218 (11)	0.0182 (11)
S1	0.94129 (4)	0.58669 (10)	0.71625 (2)	0.01954 (13)	
N1	0.77696 (15)	0.4902 (3)	0.64754 (7)	0.0186 (4)	
N2	0.74281 (16)	0.7923 (4)	0.70865 (8)	0.0219 (4)	
N3	0.80239 (15)	0.9247 (4)	0.75009 (7)	0.0219 (4)	
C1	0.85169 (18)	−0.1224 (4)	0.50103 (9)	0.0188 (4)	
C2	0.91130 (18)	−0.1876 (4)	0.54836 (9)	0.0206 (5)	
H2	0.9528	−0.3308	0.5507	0.025*	
C3	0.90938 (18)	−0.0397 (4)	0.59242 (9)	0.0192 (4)	
H3	0.9493	−0.0842	0.6252	0.023*	
C4	0.84970 (17)	0.1732 (4)	0.58934 (8)	0.0173 (4)	
C5	0.79033 (19)	0.2330 (4)	0.54094 (9)	0.0197 (5)	
H5	0.7492	0.3767	0.5382	0.024*	
C6	0.79050 (19)	0.0856 (4)	0.49681 (9)	0.0209 (5)	
H6	0.7492	0.1267	0.4642	0.025*	
C7	0.86033 (18)	0.3344 (4)	0.63585 (9)	0.0180 (4)	
C8	0.95119 (19)	0.3650 (4)	0.66889 (9)	0.0194 (5)	
H8	1.0148	0.2744	0.6666	0.023*	
C9	0.80684 (18)	0.6404 (4)	0.69039 (8)	0.0183 (4)	
C10	0.66773 (18)	0.4827 (4)	0.62245 (8)	0.0187 (4)	
C11	0.6062 (2)	0.2806 (4)	0.62887 (9)	0.0225 (5)	
H11	0.6344	0.1517	0.6508	0.027*	
C12	0.5018 (2)	0.2708 (4)	0.60235 (10)	0.0265 (5)	
H12	0.4580	0.1343	0.6065	0.032*	
C13	0.46162 (19)	0.4581 (5)	0.57017 (10)	0.0278 (5)	
H13	0.3908	0.4490	0.5520	0.033*	
C14	0.5245 (2)	0.6597 (4)	0.56423 (9)	0.0254 (5)	
H14	0.4968	0.7879	0.5420	0.030*	
C15	0.62813 (19)	0.6735 (4)	0.59085 (9)	0.0219 (5)	
H15	0.6712	0.8117	0.5874	0.026*	
C16	0.74531 (18)	1.0603 (4)	0.77618 (9)	0.0202 (5)	
C17	0.79575 (19)	1.2288 (4)	0.81892 (9)	0.0211 (5)	
H17A	0.8308	1.3662	0.8032	0.025*	
H17B	0.8499	1.1441	0.8439	0.025*	
C18	0.70068 (19)	1.3126 (5)	0.84697 (10)	0.0270 (5)	
H18A	0.7132	1.4763	0.8616	0.032*	
H18B	0.6882	1.2020	0.8762	0.032*	
C19	0.6054 (2)	1.3094 (5)	0.80314 (10)	0.0282 (5)	
H19A	0.6050	1.4549	0.7807	0.034*	
H19B	0.5363	1.2994	0.8184	0.034*	
C20	0.62414 (19)	1.0838 (5)	0.77104 (9)	0.0248 (5)	
H20A	0.5911	0.9414	0.7859	0.030*	
H20B	0.5938	1.1032	0.7334	0.030*	

### Atomic displacement parameters (Å^2^)

**Table:**

	*U*^11^	*U*^22^	*U*^33^	*U*^12^	*U*^13^	*U*^23^
Br1	0.02681 (14)	0.0192 (2)	0.02079 (13)	0.00088 (11)	0.00395 (9)	−0.00472 (11)
Br1A	0.02681 (14)	0.0192 (2)	0.02079 (13)	0.00088 (11)	0.00395 (9)	−0.00472 (11)
S1	0.0204 (3)	0.0212 (3)	0.0163 (3)	0.0003 (2)	−0.0011 (2)	−0.00170 (19)
N1	0.0182 (9)	0.0184 (9)	0.0186 (9)	0.0008 (7)	0.0001 (7)	−0.0038 (7)
N2	0.0215 (10)	0.0251 (10)	0.0185 (9)	0.0000 (8)	−0.0002 (8)	−0.0061 (8)
N3	0.0217 (10)	0.0249 (10)	0.0182 (9)	−0.0002 (8)	−0.0008 (8)	−0.0037 (8)
C1	0.0206 (11)	0.0158 (10)	0.0205 (11)	−0.0037 (9)	0.0044 (9)	−0.0035 (8)
C2	0.0203 (11)	0.0176 (10)	0.0240 (11)	0.0003 (9)	0.0029 (9)	0.0018 (9)
C3	0.0193 (11)	0.0187 (10)	0.0195 (11)	0.0000 (9)	0.0015 (9)	0.0034 (8)
C4	0.0168 (10)	0.0182 (10)	0.0167 (10)	−0.0021 (9)	0.0017 (8)	0.0008 (8)
C5	0.0225 (11)	0.0167 (10)	0.0198 (11)	0.0035 (9)	0.0017 (9)	0.0004 (8)
C6	0.0237 (12)	0.0210 (11)	0.0172 (11)	0.0020 (9)	−0.0005 (9)	0.0007 (8)
C7	0.0207 (11)	0.0160 (10)	0.0173 (10)	0.0006 (9)	0.0027 (8)	0.0021 (8)
C8	0.0221 (11)	0.0181 (10)	0.0179 (11)	0.0011 (9)	0.0020 (9)	−0.0007 (8)
C9	0.0201 (11)	0.0183 (10)	0.0157 (10)	−0.0020 (9)	−0.0012 (9)	0.0006 (8)
C10	0.0197 (11)	0.0194 (11)	0.0171 (10)	−0.0006 (9)	0.0028 (9)	−0.0041 (8)
C11	0.0264 (12)	0.0206 (11)	0.0212 (11)	−0.0027 (10)	0.0059 (9)	−0.0005 (9)
C12	0.0235 (12)	0.0260 (12)	0.0313 (13)	−0.0069 (10)	0.0089 (10)	−0.0064 (10)
C13	0.0181 (11)	0.0361 (14)	0.0286 (13)	0.0007 (11)	0.0001 (10)	−0.0095 (10)
C14	0.0250 (12)	0.0270 (12)	0.0228 (11)	0.0070 (10)	−0.0022 (10)	−0.0020 (9)
C15	0.0226 (12)	0.0196 (11)	0.0236 (11)	−0.0012 (9)	0.0028 (9)	−0.0022 (9)
C16	0.0217 (11)	0.0207 (11)	0.0176 (11)	−0.0015 (9)	0.0002 (9)	−0.0004 (8)
C17	0.0220 (11)	0.0236 (11)	0.0174 (11)	−0.0024 (9)	0.0015 (9)	−0.0029 (8)
C18	0.0245 (12)	0.0329 (13)	0.0233 (12)	0.0004 (11)	0.0024 (10)	−0.0082 (10)
C19	0.0240 (12)	0.0339 (13)	0.0264 (12)	0.0056 (11)	0.0017 (10)	−0.0062 (10)
C20	0.0221 (12)	0.0299 (13)	0.0222 (11)	−0.0009 (10)	0.0014 (9)	−0.0056 (9)

### Geometric parameters (Å, º)

**Table:**

Br1—C1	1.903 (2)	C10—C15	1.387 (3)
Br1A—C1	1.830 (8)	C11—C12	1.397 (4)
S1—C8	1.738 (2)	C11—H11	0.9500
S1—C9	1.756 (2)	C12—C13	1.382 (4)
N1—C9	1.388 (3)	C12—H12	0.9500
N1—C7	1.415 (3)	C13—C14	1.390 (4)
N1—C10	1.437 (3)	C13—H13	0.9500
N2—C9	1.289 (3)	C14—C15	1.390 (3)
N2—N3	1.421 (3)	C14—H14	0.9500
N3—C16	1.277 (3)	C15—H15	0.9500
C1—C2	1.385 (3)	C16—C20	1.511 (3)
C1—C6	1.386 (3)	C16—C17	1.514 (3)
C2—C3	1.391 (3)	C17—C18	1.530 (3)
C2—H2	0.9500	C17—H17A	0.9900
C3—C4	1.399 (3)	C17—H17B	0.9900
C3—H3	0.9500	C18—C19	1.530 (3)
C4—C5	1.398 (3)	C18—H18A	0.9900
C4—C7	1.476 (3)	C18—H18B	0.9900
C5—C6	1.389 (3)	C19—C20	1.531 (3)
C5—H5	0.9500	C19—H19A	0.9900
C6—H6	0.9500	C19—H19B	0.9900
C7—C8	1.340 (3)	C20—H20A	0.9900
C8—H8	0.9500	C20—H20B	0.9900
C10—C11	1.384 (3)		
			
C8—S1—C9	90.38 (11)	C12—C11—H11	120.8
C9—N1—C7	113.44 (18)	C13—C12—C11	120.5 (2)
C9—N1—C10	121.16 (18)	C13—C12—H12	119.7
C7—N1—C10	125.08 (18)	C11—C12—H12	119.7
C9—N2—N3	108.28 (19)	C12—C13—C14	120.2 (2)
C16—N3—N2	114.49 (19)	C12—C13—H13	119.9
C2—C1—C6	121.5 (2)	C14—C13—H13	119.9
C2—C1—Br1A	134.4 (6)	C15—C14—C13	119.9 (2)
C6—C1—Br1A	102.0 (7)	C15—C14—H14	120.0
C2—C1—Br1	119.64 (17)	C13—C14—H14	120.0
C6—C1—Br1	118.90 (17)	C10—C15—C14	119.1 (2)
C1—C2—C3	118.9 (2)	C10—C15—H15	120.4
C1—C2—H2	120.6	C14—C15—H15	120.4
C3—C2—H2	120.6	N3—C16—C20	128.6 (2)
C2—C3—C4	121.2 (2)	N3—C16—C17	121.8 (2)
C2—C3—H3	119.4	C20—C16—C17	109.62 (19)
C4—C3—H3	119.4	C16—C17—C18	104.01 (19)
C5—C4—C3	118.4 (2)	C16—C17—H17A	111.0
C5—C4—C7	123.0 (2)	C18—C17—H17A	111.0
C3—C4—C7	118.35 (19)	C16—C17—H17B	111.0
C6—C5—C4	121.0 (2)	C18—C17—H17B	111.0
C6—C5—H5	119.5	H17A—C17—H17B	109.0
C4—C5—H5	119.5	C17—C18—C19	103.86 (19)
C1—C6—C5	119.1 (2)	C17—C18—H18A	111.0
C1—C6—H6	120.5	C19—C18—H18A	111.0
C5—C6—H6	120.5	C17—C18—H18B	111.0
C8—C7—N1	112.5 (2)	C19—C18—H18B	111.0
C8—C7—C4	124.5 (2)	H18A—C18—H18B	109.0
N1—C7—C4	122.84 (19)	C18—C19—C20	103.9 (2)
C7—C8—S1	113.45 (17)	C18—C19—H19A	111.0
C7—C8—H8	123.3	C20—C19—H19A	111.0
S1—C8—H8	123.3	C18—C19—H19B	111.0
N2—C9—N1	123.8 (2)	C20—C19—H19B	111.0
N2—C9—S1	125.86 (17)	H19A—C19—H19B	109.0
N1—C9—S1	110.27 (16)	C16—C20—C19	103.95 (19)
C11—C10—C15	121.8 (2)	C16—C20—H20A	111.0
C11—C10—N1	118.9 (2)	C19—C20—H20A	111.0
C15—C10—N1	119.3 (2)	C16—C20—H20B	111.0
C10—C11—C12	118.4 (2)	C19—C20—H20B	111.0
C10—C11—H11	120.8	H20A—C20—H20B	109.0
			
C9—N2—N3—C16	−171.4 (2)	C10—N1—C9—N2	−4.5 (3)
C6—C1—C2—C3	−0.1 (3)	C7—N1—C9—S1	0.2 (2)
Br1A—C1—C2—C3	159.7 (10)	C10—N1—C9—S1	174.06 (16)
Br1—C1—C2—C3	178.96 (16)	C8—S1—C9—N2	178.2 (2)
C1—C2—C3—C4	−0.8 (3)	C8—S1—C9—N1	−0.31 (17)
C2—C3—C4—C5	1.0 (3)	C9—N1—C10—C11	−109.5 (2)
C2—C3—C4—C7	−173.6 (2)	C7—N1—C10—C11	63.6 (3)
C3—C4—C5—C6	−0.2 (3)	C9—N1—C10—C15	73.0 (3)
C7—C4—C5—C6	174.1 (2)	C7—N1—C10—C15	−113.9 (2)
C2—C1—C6—C5	0.9 (3)	C15—C10—C11—C12	0.3 (3)
Br1A—C1—C6—C5	−164.5 (6)	N1—C10—C11—C12	−177.2 (2)
Br1—C1—C6—C5	−178.20 (17)	C10—C11—C12—C13	0.6 (3)
C4—C5—C6—C1	−0.7 (3)	C11—C12—C13—C14	−0.6 (4)
C9—N1—C7—C8	0.0 (3)	C12—C13—C14—C15	−0.2 (4)
C10—N1—C7—C8	−173.5 (2)	C11—C10—C15—C14	−1.1 (3)
C9—N1—C7—C4	−174.9 (2)	N1—C10—C15—C14	176.4 (2)
C10—N1—C7—C4	11.6 (3)	C13—C14—C15—C10	1.0 (3)
C5—C4—C7—C8	−140.5 (2)	N2—N3—C16—C20	4.6 (3)
C3—C4—C7—C8	33.8 (3)	N2—N3—C16—C17	−175.20 (19)
C5—C4—C7—N1	33.8 (3)	N3—C16—C17—C18	−168.4 (2)
C3—C4—C7—N1	−151.9 (2)	C20—C16—C17—C18	11.8 (3)
N1—C7—C8—S1	−0.3 (2)	C16—C17—C18—C19	−31.0 (2)
C4—C7—C8—S1	174.50 (17)	C17—C18—C19—C20	38.9 (3)
C9—S1—C8—C7	0.35 (18)	N3—C16—C20—C19	−167.8 (2)
N3—N2—C9—N1	−176.3 (2)	C17—C16—C20—C19	12.1 (3)
N3—N2—C9—S1	5.4 (3)	C18—C19—C20—C16	−31.2 (2)
C7—N1—C9—N2	−178.3 (2)		

### Hydrogen-bond geometry (Å, º)

**Table:**

*D*—H···*A*	*D*—H	H···*A*	*D*···*A*	*D*—H···*A*
C17—H17*B*···Br1^i^	0.99	3.03	3.828 (2)	138

Symmetry code: (i) *x*, −*y*+1/2, *z*+1/2.
